# Comparative longitudinal evaluation of DIMS, progressive addition and single-vision lenses on myopia progression, binocular and visual performance in Spanish children

**DOI:** 10.1371/journal.pone.0361471

**Published:** 2026-10-26

**Authors:** Salvador Solano-Molina, Miriam Carrillo-Pulido, Sonia Ortiz-Peregrina, Francesco Martino, Rosario G. Anera, José R. Jiménez

**Affiliations:** 1 Centro Nazaríes Especialistas, Padul, Granada, Spain; 2 Department of Optics, Laboratory of Vision Sciences and Applications, University of Granada, Granada, Spain; The Ohio State University, UNITED STATES OF AMERICA

## Abstract

An 18-month prospective, non-randomized comparative observational study evaluated the effects of different optical corrections on myopia progression, binocular vision, and visual performance in Spanish children. Forty-five myopic participants aged 9–14 years were allocated to single-vision lenses (SV), low-addition progressive addition lenses (PALs), or defocus-incorporated multiple segments lenses (DIMS). Assessments included spherical equivalent (SE) and axial length (AL), comprehensive accommodative function evaluated by measuring amplitude of accommodation, positive and negative relative accommodation, accommodative facility, and accommodative response, and binocular function evaluated by measuring phorias, fusional vergences, and stereopsis. In addition, contrast sensitivity (CS), visual disturbance index (VDI), and subjective visual quality were assessed at baseline and at 6, 12, and 18 months. After 18 months, SE progression was −0.59 D for SV, −0.51 D for PALs, and −0.30 D for DIMS, corresponding to a 49% reduction with DIMS compared to SV. AL increased by 0.24, 0.27, and 0.23 millimeters, respectively, although inter-group differences were not significant for AL. Accommodative and binocular functions, visual acuity, CS, and VDI remained stable, while distance stereopsis improved in the DIMS group. These findings suggest an association between DIMS lens wear and reduced myopia progression compared with SV lenses in this cohort, without clinically relevant differences in accommodative or binocular function, visual acuity, or subjective visual quality.

## 1. Introduction

The high percentage of the global population affected by myopia, together with projections indicating a substantial increase in its prevalence in the coming decades, makes myopia and the control of its progression highly relevant topics in epidemiology and public health research [1–3]. Treatments and techniques aimed at slowing myopia progression in children and young adults are of particular importance in optometric practice, and various approaches and strategies have been proposed to attempt to reduce such progression [4,5], including the use of ophthalmic lenses for myopia control.

In recent years, there has been an expansion in the development of ophthalmic lenses designed to induce peripheral myopic defocus and slow myopia progression, such as DIMS (Defocus Incorporated Multiple Segments, MiyoSmart®, Hoya) and Stellest® (Essilor) lenses [6,7]. Other spectacle lens technologies have also emerged, including peripheral defocus lenses based on cylindrical refractive patterns (MyoCare®, ZEISS) [8] and diffusion optics technology [9,10]. Although peripheral myopic defocus has traditionally been proposed as the primary mechanism underlying these lens designs, alternative mechanisms, including modulation of peripheral contrast signals, have also been suggested [9,10].

Progressive addition lenses (PALs) were previously introduced with the aim of reducing accommodative demand during near work. Although reducing accommodative demand has been proposed to decrease hyperopic retinal defocus and contribute to slowing myopia progression, the underlying mechanism remains under debate [10–13]. While PALs are no longer considered a first-line treatment for myopia control, they were among the most extensively studied spectacle-lens interventions before the introduction of peripheral myopic defocus spectacle lenses and are therefore included in the present study as a historical comparator [14].

Among spectacle lenses for myopia control, DIMS lenses are widely used. Most studies, as is the case with other myopia control lenses, have focused primarily on outcomes directly related to myopia progression, such as axial length and spherical equivalent refraction [15–17]. However, relatively few studies have investigated the effects of DIMS lenses on other visual functions, including binocular vision and visual performance [15,18]. Although the primary objective of these lenses is to slow myopia progression, it is also important to determine whether their use is associated with changes in other aspects of visual function. Recently, Yahaya et al. [18] evaluated binocular vision and accommodation in myopic children wearing DIMS lenses for two years by assessing stereopsis, phorias, fusional vergences, and accommodative response.

Beyond binocular vision, other aspects of visual performance, such as contrast sensitivity (CS), also warrant investigation. Contrast sensitivity assessment provides valuable information about the ability to detect contrast across different spatial frequencies; however, only a limited number of studies have evaluated the impact of DIMS lenses on this parameter [19,20]. Notably, these studies were conducted under laboratory conditions following short-term lens wear, rather than in habitual DIMS lens wearers using the lenses in daily life over extended periods.

Furthermore, it is well established that certain emmetropization procedures, including both surgical and optical interventions, may be associated with reductions in visual quality, with patients reporting symptoms such as halos, glare, starbursts, blur, and ghost images [21–24]. These effects are theoretically more pronounced with multifocal optical correction systems or lens designs incorporating multiple optical powers across different zones, such as DIMS lenses. McCullough et al. [25] evaluated visual quality in children wearing DIMS lenses using questionnaires that assessed symptoms including blur and ghost images. To complement these subjective assessments and reduce potential bias, software-based tools capable of generating a Visual Disturbance Index (VDI) enable the quantitative evaluation of visual symptoms [23].

The present study was designed to evaluate myopia progression control and visual performance, as well as accommodative and binocular visual function, in myopic children wearing different ophthalmic lens designs (DIMS, PALs, and single-vision lenses), allowing a descriptive comparison of outcomes across lens groups. Contrast sensitivity was measured to assess visual performance and quality, and visual disturbances such as halos, glare, and starbursts were evaluated using VDI, together with standardized questionnaires commonly used in this field of research.

This study provides a novel and comprehensive observational assessment of myopia progression control, binocular and visual function, and visual performance in a cohort of Spanish myopic children using different corrective lens modalities.

## 2. Methodology

A prospective, non-randomized, longitudinal comparative observational study was conducted over an 18-month period from September 2023 to June 2025 at the Centro Óptico Nazaríes (Padúl, Granada, Spain), in collaboration with the University of Granada. The study was designed to evaluate myopia progression, as well as visual, accommodative, and binocular functions, in myopic children wearing different ophthalmic lens designs.

### 2.1. Participants

Participants were allocated to one of three optical correction groups: DIMS lenses, low-addition progressive lenses (PALs), or single-vision (SV) ophthalmic lenses, which served as the control group. A total of 45 participants were included and distributed into three groups according to the type of lens worn: SV (n = 16), DIMS (n = 16), and PALs (n = 13). Group allocation was determined by participant and guardian preference and treatment availability under the supervision of the principal investigator. The study was non-randomized due to ethical and practical constraints related to treatment preference and clinical feasibility. The flow of participants through the study is shown in Fig 1.

**Fig 1:**
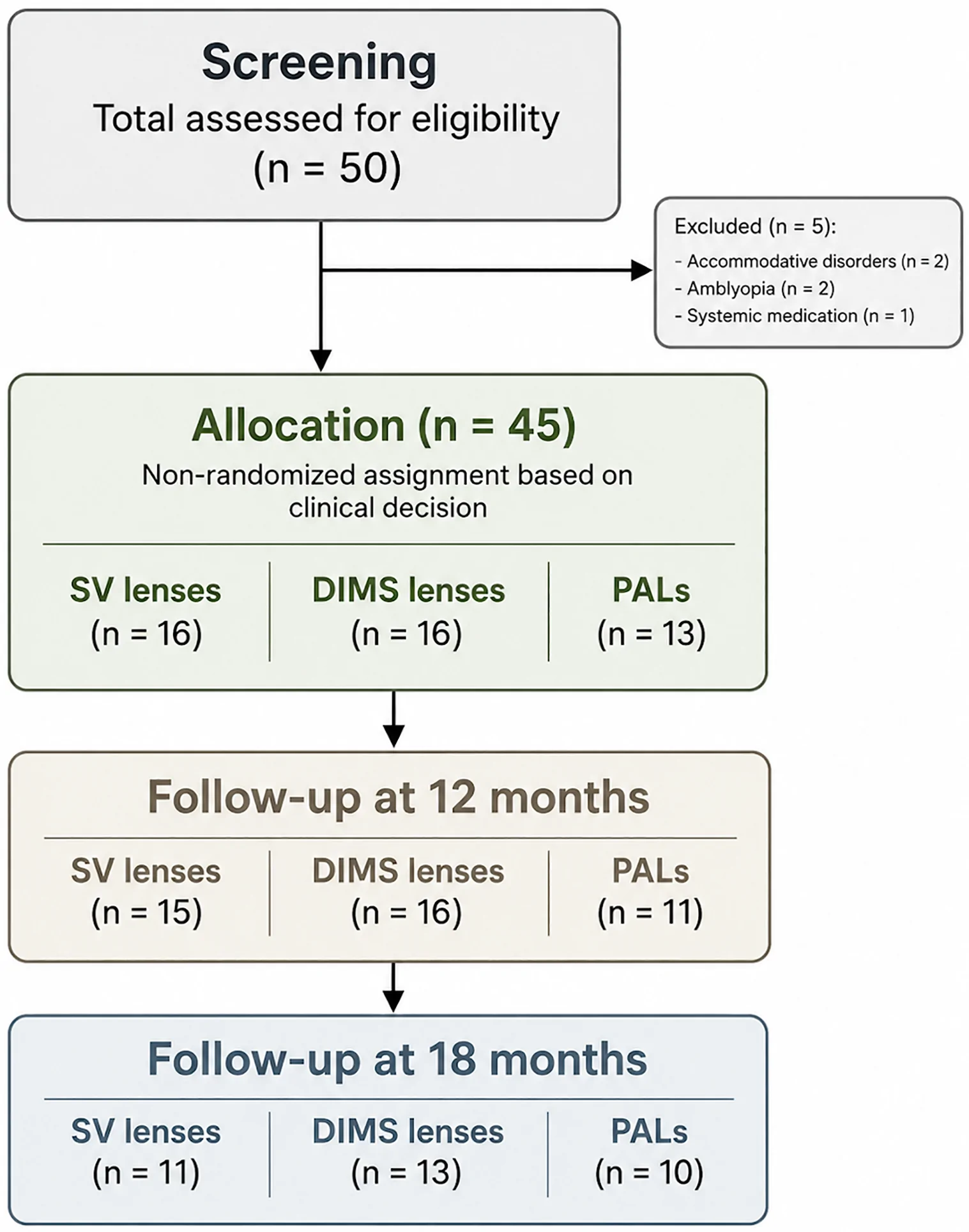
Participant Flow Diagram of Study Enrollment, Allocation, and Follow-up.

Inclusion criteria comprised children aged between 9 and 14 years with progressing myopia, spherical error or spherical equivalent greater than or equal to −0.75 D, astigmatism of 1.00 D or less, and a maximum anisometropia of 1.50 D. For the purposes of this study, progressive myopia was defined as a myopic shift in spherical equivalent refraction greater than 0.50 D over 12 months, based on the IMI Clinical Management Guidelines Report [26]. Exclusion criteria included previous use of rigid gas-permeable contact lenses or any myopia control intervention, ocular pathology, binocular vision abnormalities (e.g., manifest strabismus or amblyopia), use of medications affecting accommodation or pupil size, corneal abnormalities, or systemic diseases potentially affecting visual development.

Each participant underwent a comprehensive ophthalmic examination, including assessment of refractive error, visual acuity at multiple distances, axial length, accommodative and binocular vision parameters, and visual performance. Assessments were performed at four scheduled visits: baseline, 6, 12, and 18 months, following the same clinical protocol at each time point. At the 12-month visit, two validated questionnaires were administered to evaluate subjective visual function: the Quality of Vision (QoV) questionnaire [27,28] to assess perceived everyday visual quality, and the Conlon questionnaire [29] to determine the presence and frequency of symptoms related to binocular vision and visual strain. At the 12-month follow-up visit, participants completed Spanish translations of both questionnaires through a Google Forms survey, with assistance from the examiner when required. The study was approved by the Ethics Committee of the University of Granada (921/CCEIH/2019), and all procedures adhered to the principles of the Declaration of Helsinki. Written informed consent was obtained from parents or legal guardians, along with assent from the children prior to study participation. Group allocation was based on participant and guardian preference and availability, and it was supervised by the principal investigator.

Participants received the optical correction corresponding to their assigned group. DIMS lenses (MiYOSMART®, Hoya) feature a central zone for distance refractive correction and a peripheral zone with multiple segments that generate a positive addition of +3.50 D, designed to slow myopia progression [6,7]. PALs (Cosmolit 0.8, Rodenstock®) incorporate an aspheric progressive addition design of +0.80 D, intended to reduce accommodative demand at near, a mechanism that may also contribute to reducing myopia progression [11,30,31]. Single-Vision (SV) lenses corrected only distance refractive error and served as the control group. All participants were instructed to wear their lenses for most of their waking hours, ensuring full optical exposure to the effects of each lens design.

### 2.2. Visual Examination Tests

#### 2.2.1. Myopia control.

To characterize parameters related to myopia progression, the MYAH topographer (Topcon, Japan) was used. Ocular axial length (AL, mm) was recorded using integrated optical biometry, and keratometry readings of the flattest and steepest corneal meridians (K′ and K, D) were obtained. Refractive error was assessed using non-cycloplegic autorefraction with an autorefractometer (Nidek Tonoref III, NIDEK, Japan) and retinoscopy (Welch Allyn Elite, Welch Allyn, USA), followed by subjective refraction. The spherical equivalent (SE, D) was calculated as the spherical power plus half of the cylindrical power (SE = sphere + cylinder/2), with refractive errors expressed in negative-cylinder notation. These measurements allowed monitoring of changes in refractive error and ocular morphology throughout the study period.

#### 2.2.2. Visual function.

Visual function evaluation was performed using the CSV1000P optotype (Rodenstock®) under controlled photopic lighting conditions (85 cd/m²), with standardized optotype contrast [32]. Distance and near visual acuity were measured under monocular and binocular conditions, recording the following parameters: monocular distance visual acuity with correction (MDVA-CC, 6 m), binocular distance visual acuity with correction (BDVA-CC, 6 m) and binocular near visual acuity with correction (BNVA-CC, 40 cm).

Additionally, contrast sensitivity (CS) was evaluated monocularly and binocularly using the CSV1000P at spatial frequencies of 1.5, 3, 6, 12, and 18 cycles per degree (cpd). The visual disturbance index (VDI) was measured at each visit under monocular and binocular low-light conditions using Halo v1.0 software (Vision Sciences and Applications Laboratory, Universidad de Granada, Spain) [23]. The VDI reflects the proportion of peripheral stimuli not detected relative to the total presented, with values close to 1 indicating greater visual disturbance and reduced discrimination capacity. This index quantifies the potential effects of visual symptoms such as halos, glare, starbursts, and residual images, as discrimination ability decreases in their presence. This test has been widely used in studies involving various experiments and emmetropization techniques where these visual symptoms may occur [23,24].

At the 12-month follow-up, two validated questionnaires were administered for the subjective evaluation of visual function. The Quality of Vision (QoV) questionnaire [27], comprising 30 items, assesses ten common visual symptoms, including glare, halos, starbursts, and blurred or fluctuating vision. Each symptom is rated across three dimensions—frequency, severity, and degree of bother—and a mean score per symptom is calculated, where values near 0 indicate better visual quality and higher values indicate greater discomfort. Results were used to compare the DIMS, SV, and PALs groups. Although the QoV includes items on glare, starbursts, and halos, we also performed an objective measurement of a VDI based on stimulus discrimination, providing a quantitative assessment of these visual disturbances. McCullough et al. [25] previously administered a similar questionnaire to DIMS lens wearers and reported discrepancies between subjective responses and objective visual acuity tests, highlighting the limitations of assessments based solely on questionnaires.

The Conlon questionnaire [29], comprising 23 items, identifies symptoms associated with visual stress. Total scores and five clinical subscales—asthenopia, headache, reading difficulties, perceptual distortions, and brightness/glare—were analyzed, allowing a multidimensional evaluation of visual symptoms.

#### 2.2.3. Accommodation tests.

Accommodation testing was performed to evaluate multiple components of the accommodative system, including amplitude, response, and facility of accommodation, under controlled conditions. All participants were optimally corrected for distance vision. Near vision testing was conducted using a CSV1000P near optotype, with selection of a line immediately below the participant’s near visual acuity at 40 cm. Two measurements were obtained for each parameter, and the mean value was calculated. Participants were instructed to maintain the target clear, with the endpoint defined as the first sustained blur that could not be cleared despite effort.

Amplitude of accommodation (AA) was measured both monocularly and binocularly using the push-up (Donders) method, by gradually moving the target closer until blur was reported and then away until clarity was restored. AA was calculated in diopters as the reciprocal of the distance to the blur point. Positive relative accommodation (PRA) and negative relative accommodation (NRA) were assessed monocularly using a phoropter, with lenses added in 0.25 D increments until sustained blur was reached. Accommodative facility (AF) was determined using ±2.00 D flippers, recording the number of complete cycles per minute. Finally, accommodative response (AR) was measured using binocular dynamic retinoscopy, noting the lens power required to neutralize the retinoscopic reflex.

#### 2.2.4. Binocular vision tests.

Binocular function was assessed under participants’ habitual optical correction by measuring phorias, fusional vergences, and stereopsis. Phorias were recorded at distance (6 m) and near (40 cm) and expressed as distance horizontal phoria (DHP), distance vertical phoria (DVP), near horizontal phoria (NHP), and near vertical phoria (NVP), using the Maddox and Thorington methods. Values were reported in prism diopters (Δ), with positive values indicating esophoria or hyperphoria and negative values indicating exophoria or hypophoria.

Fusional vergences were measured using a prism bar, with prism power gradually increased to determine the blur, break, and recovery points for both positive fusional vergence (PFV, base-out) and negative fusional vergence (NFV, base-in) at distance and near. Distance stereopsis (D-Stereo) was assessed at 5 m using the Rodenstock CSV1000P optotype screen, while near stereopsis (N-Stereo) was evaluated at 40 cm using the Randot Test. Stereopsis results were expressed in seconds of arc, recording the minimum disparity that participants were able to resolve consistently.

### 2.3. Statistical analysis

Statistical analyses were conducted using Python (v. 3.13.2) within the Jupyter Notebook environment. Normality of quantitative variables was assessed using the Shapiro–Wilk test in the total sample and by group (n < 50). Parametric or non-parametric tests were applied according to the results, with a significance level set at p < 0.05.

Demographic and clinical variables were described using mean ± standard deviation, and minimum and maximum values when appropriate. Inter-group comparisons were conducted using one-way ANOVA for variables meeting assumptions of normality and homogeneity of variances, followed by Tukey post hoc tests. For variables that did not meet these assumptions, the Kruskal–Wallis test was applied, with post hoc analysis using Dunn’s test and Bonferroni correction. Categorical variables were analyzed using the chi-square (χ²) test.

Longitudinal intra-group analysis followed two approaches: comparisons between baseline and 18 months using paired Student’s t-test for normally distributed variables or the Wilcoxon signed-rank test for non-normally distributed variables, and analysis across all visits (baseline, 6, 12, and 18 months) using the Friedman test to detect significant changes over time. When significant Friedman results were obtained, post hoc pairwise comparisons were performed using the Wilcoxon signed-rank test with Bonferroni correction. In cases with missing data at any follow-up visit, a complete-case paired analysis (listwise deletion) was applied, excluding participants from the corresponding longitudinal comparison whenever data were not available at either of the two time points.

QoV and Conlon questionnaires were analyzed by calculating the mean and standard deviation of each symptom or subscale, grouping items into composite variables based on their average scores. Internal consistency was evaluated using Cronbach’s alpha, considering individual groups and the overall sample, with thresholds defined as acceptable (α ≥ 0.70), good (α ≥ 0.80), and excellent (α ≥ 0.90).

## 3. Results

A total of 45 participants were included and distributed into three treatment groups: SV (n = 16), DIMS (n = 16), and PALs (n = 13). Baseline age differed significantly among groups (p = 0.002), according to Dunn’s post hoc analysis. The DIMS group was younger than SV (p = 0.043) and PALs (p = 0.001), whereas no significant differences were observed between SV and PALs. Mean ages were 9.94 ± 2.77 years for DIMS, 12.50 ± 2.76 years for SV, and 13.92 ± 2.40 years for PALs. Sex distribution was comparable across groups (χ² = 0.652, p = 0.723).

Regarding baseline ocular variables, SE showed significant differences among groups (p = 0.036), mainly attributable to greater myopia in the PALs group compared with SV, according to Dunn’s post hoc test (p = 0.048). AL also differed significantly between groups (p = 0.005), being slightly greater in the PALs group, consistent with their higher SE. No relevant differences were observed at baseline in other visual function variables, including visual acuity (near, distance, binocular, monocular) or corneal curvature radii (K´ and K). Baseline participant characteristics are summarized in Table 1.

**Table 1:** Baseline ocular characteristics of participants by Myopia control treatment.

Variable	Group	Mean ± SD	Min–Max
SE (D)	SV	−1.547 ± 0.877	−4.125 – −0.125
	DIMS	−1.883 ± 1.502	−6.000 – −0.500
	PALs	−2.740 ± 1.415	−5.625 – −0.750
UCVA-MC (logMAR)	SV	0.003 ± 0.004	0.000–0.009
	DIMS	0.004 ± 0.006	0.000–0.018
	PALs	0.031 ± 0.099	0.000–0.398
BCVA-DC (logMAR)	SV	0.000 ± 0.000	0.000–0.000
	DIMS	0.001 ± 0.002	0.000–0.009
	PALs	0.021 ± 0.071	0.000–0.284
BCVA-NC (logMAR)	SV	0.000 ± 0.000	0.000–0.000
	DIMS	0.000 ± 0.000	0.000–0.000
	PALs	0.000 ± 0.000	0.000–0.000
AL (mm)	SV	23.737 ± 0.879	21.680–25.290
	DIMS	23.660 ± 0.913	22.670–25.320
	PALs	24.676 ± 0.738	23.495–26.255
K´(mm)	SV	7.757 ± 0.306	7.150–8.280
	DIMS	7.780 ± 0.283	7.270–8.360
	PALs	7.936 ± 0.204	7.530–8.365
K (mm)	SV	7.589 ± 0.306	7.070–8.190
	DIMS	7.619 ± 0.307	7.190–8.190
	PALs	7.727 ± 0.214	7.385–8.050

***Abbreviations:*** *SE, Spherical Equivalent (D); UCVA-MC, Monocular Distance Visual Acuity with Correction (logMAR, 6 m); BCVA-DC, Binocular Distance Visual Acuity with Correction (logMAR, 6 m); BCVA-NC, Binocular Near Visual Acuity with Correction (logMAR, 40 cm); AL, Axial Length (mm); K, Keratometry Flat Meridian (mm); K´, Keratometry Steep Meridian (mm).*

### 3.1. Myopia control

Myopia progression, evaluated as cumulative changes in SE is presented in Table 2. At 18 months, mean SE progression was −0.59 D in the SV group, −0.51 D in the PALs group, and −0.30 D in the DIMS group.

**Table 2:** Longitudinal progression of spherical equivalent (SE) across follow-up visits.

Group	Δ6M (mean ± SD, N)	Δ12M (mean ± SD, N)	Δ18M (mean ± SD, N)
SV	−0.31 ± 0.31 (N = 16)	−0.48 ± 0.46 (N = 15)	−0.59 ± 0.62 (N = 11)
DIMS	−0.16 ± 0.42 (N = 16)	−0.20 ± 0.32 (N = 16)	−0.30 ± 0.36 (N = 13)
PALs	−0.42 ± 0.44 (N = 13)	−0.40 ± 0.43 (N = 11)	−0.51 ± 0.63 (N = 10)

***Abbreviations:*** *SV, single-vision; DIMS, Defocus Incorporated Multiple Segments lenses; PALs, Progressive Addition Lenses. Cumulative changes were calculated relative to baseline visit (Δ = change from baseline).*

Longitudinal analyses of absolute SE values are provided in S1 Table. Significant changes over time were observed in the SV group (χ² = 14.87, p = 0.002), DIMS group (χ² = 8.26, p = 0.041), and PALs group (χ² = 13.22, p = 0.004). Post hoc comparisons identified significant differences between baseline and the 6-month visit in the SV (p = 0.0117) and PALs (p = 0.0059) groups, while no significant pairwise differences between consecutive visits were observed in the DIMS group.

Similarly, cumulative changes in AL are presented in Table 3. At 18 months, mean axial elongation was 0.32 mm in the SV group, 0.23 mm in the DIMS group, and 0.27 mm in the PALs group.

**Table 3:** Longitudinal progression of axial length (AL) across follow-up visits.

Group	Δ6M (mean ± SD, N)	Δ12M (mean ± SD, N)	Δ18M (mean ± SD, N)
SV	0.17 ± 0.29 (N = 16)	0.22 ± 0.32 (N = 15)	0.32 ± 0.42 (N = 11)
DIMS	0.11 ± 0.17 (N = 16)	0.15 ± 0.19 (N = 16)	0.23 ± 0.28 (N = 13)
PALs	0.12 ± 0.10 (N = 13)	0.20 ± 0.16 (N = 11)	0.27 ± 0.21 (N = 10)

***Abbreviations:*** *SV, single-vision; DIMS, Defocus Incorporated Multiple Segments lenses; PALs, Progressive Addition Lenses. Cumulative changes were calculated relative to baseline visit (Δ = change from baseline)*

Longitudinal analyses of absolute AL values are provided in S2 Table. Significant changes over time were observed in the SV group (χ² = 19.35, p < 0.001), DIMS group (χ² = 12.95, p = 0.005), and PALs group (χ² = 22.44, p < 0.001). Post hoc comparisons identified a significant difference between the 12- and 18-month visits in the SV group. In the PALs group, significant differences were observed between the 6- and 12-month visits and between the 12- and 18-month visits. No significant pairwise differences between consecutive visits were observed in the DIMS group.

### 3.2. Visual function

Throughout the follow-up period, visual acuity remained stable across all participants, irrespective of the myopia control intervention. Assessments included both corrected binocular and monocular visual acuity. No statistically significant differences were detected between groups at baseline or at the conclusion of the study. At 18 months, mean binocular visual acuity was 0.000 ± 0.000 logMAR in the SV, DIMS, and PAL groups, with no significant change relative to baseline measurements.

CS assessment allowed analysis of visual function beyond conventional visual acuity, reflecting the subjects’ ability to detect luminance differences at low contrast. During follow-up, binocular CS remained generally stable across the three groups, with no clinically relevant changes at most spatial frequencies. The DIMS group showed longitudinal improvements at 1.5 cpd (p = 0.039) and 18 cpd (p = 0.008), whereas SV showed a significant increase only at 18 cpd (p = 0.016), and PALs showed no significant changes. Baseline comparison using the Kruskal–Wallis test revealed a difference at 1.5 cpd (p = 0.008), which disappeared at subsequent visits, indicating that CS was not adversely affected by treatment.

Regarding binocular VDI, baseline mean values were 0.24 ± 0.19 (SV), 0.26 ± 0.19 (DIMS), and 0.27 ± 0.21 (PALs), with no significant differences between groups (p = 0.948). At 18 months, values remained stable at 0.18 ± 0.13, 0.22 ± 0.27, and 0.15 ± 0.14 for SV, DIMS, and PALs, respectively, again with no significant intergroup differences (p = 0.820).

At 12 months, two validated questionnaires were administered to assess subjective perception of visual function: Quality of Vision (QoV) and the Conlon questionnaire. Both demonstrated excellent overall internal consistency (QoV α = 0.936; Conlon α = 0.942), supporting their reliability for between-group comparisons.

QoV symptom-specific results are presented in Table 4.8. Overall, symptom perception was higher in PALs, followed by SV and DIMS, although no statistically significant differences were observed for any individual item. Global scores showed the same trend (DIMS 0.67 ± 0.41; SV 0.77 ± 0.39; PALs 0.98 ± 0.51; p = 0.208). Conlon Questionnaire subscale scores are shown in Table 4.9, with no significant differences between groups. Global mean scores were 0.58 ± 0.41 for SV, 0.86 ± 0.73 for PALs, and 0.54 ± 0.41 for DIMS (Kruskal–Wallis, p = 0.452), indicating greater symptomatology in the PALs group, although without statistically significant differences.

### 3.3. Accommodative function

Table 4 presents the longitudinal evolution of accommodative parameters in myopic children according to lens group (SV, DIMS, and PALs) at baseline and at 6, 12, and 18 months. Mean values and standard deviations are reported for each variable and group, with repeated-measures analyses across all visits showing only minor temporal changes.

**Table 4:** Longitudinal evolution of accommodative variables in myopic children by lens group, with p-values comparing baseline versus 18 months.

Variable	Group	Basal	6m	12m	18m	Longitudinal p-value
AA-M	SV	8.41 ± 5.82	6.11 ± 2.05	6.80 ± 2.89	8.07 ± 4.05	0.507
	DIMS	7.53 ± 4.93	5.32 ± 2.14	6.06 ± 3.82	6.92 ± 3.92	0.053
	PAL	8.27 ± 4.71	5.87 ± 2.67	5.75 ± 2.73	7.78 ± 2.47	**0.006**
AF-M	SV	7.61 ± 2.16	12.02 ± 3.58	11.00 ± 3.28	10.93 ± 3.44	**0.001**
	DIMS	6.88 ± 3.80	11.55 ± 3.83	12.39 ± 4.33	10.96 ± 4.94	0.072
	PAL	8.67 ± 2.83	12.08 ± 3.39	12.64 ± 4.54	13.05 ± 3.02	**0.006**
AR	SV	0.79 ± 0.26	0.83 ± 0.31	0.92 ± 0.31	0.84 ± 0.26	0.960
	DIMS	0.83 ± 0.23	0.81 ± 0.14	0.79 ± 0.20	0.83 ± 0.24	0.989
	PAL	0.87 ± 0.25	0.95 ± 0.24	0.91 ± 0.28	0.80 ± 0.20	0.557
PRA	SV	−2.22 ± 1.56	−3.31 ± 2.45	−3.37 ± 2.50	−2.94 ± 2.27	0.768
	DIMS	−2.81 ± 2.26	−3.83 ± 3.12	−3.90 ± 3.33	−3.74 ± 2.52	0.393
	PAL	−3.22 ± 2.07	−4.41 ± 2.71	−5.35 ± 3.21	−4.25 ± 2.66	0.084
NRA	SV	2.65 ± 0.52	2.67 ± 0.32	2.54 ± 0.45	2.56 ± 0.57	0.411
	DIMS	2.61 ± 0.84	2.68 ± 0.48	2.66 ± 0.36	2.58 ± 0.40	0.983
	PAL	2.56 ± 0.67	3.01 ± 0.67	2.68 ± 0.82	2.59 ± 0.93	0.263
AA-B	SV	7.39 ± 4.32	5.30 ± 2.13	5.75 ± 2.29	7.82 ± 5.07	0.361
	DIMS	6.80 ± 4.84	4.78 ± 2.08	5.61 ± 3.63	6.65 ± 3.82	**0.013**
	PAL	7.91 ± 3.65	5.44 ± 2.71	4.82 ± 1.87	7.23 ± 2.69	**0.005**
AF-B	SV	9.48 ± 2.98	11.63 ± 4.24	10.58 ± 3.62	9.80 ± 3.41	0.384
	DIMS	6.73 ± 2.47	10.25 ± 3.62	9.45 ± 4.30	9.40 ± 3.70	0.260
	PAL	10.02 ± 4.27	11.71 ± 4.72	11.30 ± 4.33	10.38 ± 4.99	0.549

***Abbreviations:*** *AA-M, monocular amplitude of accommodation (D); AA-B, binocular amplitude of accommodation (D); PRA, positive relative accommodation (D); NRA, negative relative accommodation (D); AF-M, monocular accommodative facility (cycles per minute); AF-B, binocular accommodative facility (cycles per minute); AR, accommodative response (D).*

Between-group comparisons at each time point indicated that only AF-B differed significantly at baseline (p = 0.022), whereas no significant differences were observed at 18 months, suggesting similar patterns of accommodative function and binocular vision across lens designs. Within-group analyses revealed significant changes over time in only five conditions at the 18-month follow-up (Table 4).

### 3.4. Binocular vision

Binocular vision was evaluated through horizontal and vertical phorias, positive and negative fusional vergences, and distance and near stereopsis. Analysis of phorias revealed no significant changes over time for any measured variable (DHP, DVP, NHP, NVP) within any of the lens groups. Similarly, no significant differences were observed between groups at any follow-up visit (p > 0.05), with phorias remaining relatively stable over the 18-month period across all lens types. (Table 5).

**Table 5:** Longitudinal evolution of horizontal and vertical phorias at distance and near according to lens type in myopic children.

Variable	Group	Basal	6m	12m	18m	Longitudinal p-value
DHP	SV	1.08 ± 5.20	2.47 ± 3.75	1.17 ± 3.92	1.55 ± 4.62	0.531
	DIMS	3.03 ± 4.82	3.30 ± 5.35	3.33 ± 4.34	3.33 ± 5.54	0.985
	PAL	−0.44 ± 3.86	1.06 ± 5.21	1.82 ± 4.94	0.73 ± 4.55	0.234
DVP	SV	−0.36 ± 2.18	−1.27 ± 2.11	−1.25 ± 2.21	−0.59 ± 1.20	0.663
	DIMS	−1.38 ± 2.86	−0.97 ± 1.88	−0.61 ± 1.55	0.04 ± 0.66	0.986
	PAL	−0.67 ± 1.50	−0.54 ± 2.10	−1.21 ± 2.35	−0.35 ± 1.03	0.726
NHP	SV	−1.16 ± 7.87	1.19 ± 4.20	−0.05 ± 3.95	−0.02 ± 6.28	0.848
	DIMS	−0.89 ± 4.40	−0.20 ± 4.41	1.08 ± 3.14	0.19 ± 2.85	0.469
	PAL	0.90 ± 5.04	−1.92 ± 5.81	0.66 ± 5.27	−0.50 ± 3.46	0.165
NVP	SV	−0.08 ± 3.16	−0.95 ± 2.92	−0.58 ± 1.29	−0.68 ± 1.94	0.976
	DIMS	−1.11 ± 3.05	0.09 ± 0.38	0.09 ± 0.38	−0.31 ± 1.23	0.696
	PAL	−0.85 ± 2.34	0.04 ± 0.52	−0.61 ± 1.60	−0.15 ± 0.47	0.307

***Abbreviations:*** *DHP, Distance Horizontal Phoria, DVP, Distance Vertical Phoria, NHP, Near Horizontal Phoria, NVP, Near Vertical Phoria.*

***Notes:*** *Values are presented as mean ± standard deviation in prism diopters (Δ). p-values correspond to longitudinal analyses across visits. Positive values indicate esophoria/hyperphoria, negative values indicate exophoria/hypophoria.*

Positive and negative vergences were subsequently analyzed (Table 6). At baseline, significant differences between groups were observed for negative recovery at distance (p = 0.013) and near (p = 0.018); however, these differences were not maintained longitudinally, suggesting limited clinical relevance.

**Table 6:** Longitudinal evolution of horizontal and vertical fusional vergences by lens type and viewing distance in myopic children.

Variable	Group	Basal (BP / REC)	6 months (BP /REC)	12 months (BP/ REC)	18 months (BP / REC)
PFV Distance	SV	21.09 ± 7.97 / 11.25 ± 5.18	16.17 ± 9.02 / 9.23 ± 4.62	15.80 ± 8.95 / 10.50 ± 6.52	18.46 ± 9.77 / 13.82 ± 8.32
	DIMS	19.81 ± 10.80 / 10.25 ± 6.40	16.25 ± 9.41 / 9.94 ± 5.70	15.03 ± 6.41 / 10.47 ± 6.27	15.15 ± 7.72 / 9.73 ± 6.64
	PAL	23.92 ± 10.46 / 13.08 ± 4.70	21.50 ± 7.14 / 14.58 ± 4.68	23.77 ± 8.18 / 16.41 ± 5.70	22.25 ± 9.55 / 16.60 ± 4.99
NFV Distance	SV	13.00 ± 5.83 / 5.69 ± 3.36	7.50 ± 6.07 / 4.11 ± 3.43	8.00 ± 4.19 / 5.43 ± 3.66	8.46 ± 4.03 / 4.55 ± 2.70
	DIMS	9.44 ± 6.66 / 3.50 ± 3.02	6.75 ± 3.30 / 3.34 ± 2.39	6.19 ± 1.52 / 2.69 ± 1.08	6.85 ± 1.91 / 3.50 ± 1.94
	PAL	12.31 ± 5.98 / 5.19 ± 2.43	8.00 ± 3.81 / 4.77 ± 2.62	7.77 ± 3.78 / 5.73 ± 3.20	8.30 ± 2.91 / 5.90 ± 2.51
PFV Near	SV	26.92 ± 12.53 / 15.90 ± 8.56	18.06 ± 7.88 / 12.09 ± 4.99	17.30 ± 6.86 / 11.67 ± 4.44	20.73 ± 8.94 / 13.05 ± 4.20
	DIMS	24.84 ± 11.10 / 13.06 ± 7.40	19.28 ± 8.14 / 11.72 ± 6.99	20.94 ± 7.29 / 13.59 ± 5.57	19.08 ± 6.78 / 13.39 ± 5.12
	PAL	28.90 ± 9.94 / 18.32 ± 7.70	24.27 ± 9.23 / 15.85 ± 5.90	28.59 ± 8.80 / 20.36 ± 5.09	26.75 ± 7.61 / 20.40 ± 4.70
NFV Near	SV	16.00 ± 11.15 / 7.00 ± 4.03	12.50 ± 4.37 / 8.19 ± 4.04	12.00 ± 5.89 / 6.60 ± 4.24	11.46 ± 4.70 / 7.36 ± 3.75
	DIMS	14.16 ± 9.93 / 5.72 ± 4.57	9.50 ± 3.27 / 4.94 ± 3.30	11.06 ± 4.33 / 5.94 ± 3.89	10.92 ± 4.77 / 7.08 ± 4.27
	PAL	20.95 ± 11.96 / 10.13 ± 4.79	12.46 ± 3.36 / 8.31 ± 3.30	11.73 ± 7.09 / 7.55 ± 3.56	15.10 ± 3.64 / 10.00 ± 4.40

***Abbreviations:*** *PFV, Positive Fusional Vergence; NFV, Negative Fusional Vergence; BP, break point; REC, recovery point; Distance, measurements taken at far viewing distance; Near, measurements taken at near viewing distance.*

***Notes:*** *Values are mean ± SD in prism diopters (Δ). Positive values indicate convergence/elevation, negative values divergence/depression. BP is the prism power at blur or diplopia onset; REC is the prism power at restoration of single binocular vision.*

At 18 months, isolated differences were detected in the SV group for negative break (p < 0.001) and recovery (p = 0.006) at distance, and in the DIMS group for negative break (p = 0.002) at near. These longitudinal changes remained within expected physiological ranges and should be interpreted with caution regarding clinical relevance.

Finally, Table 7 shows the evolution of distance and near stereopsis, with no significant differences between groups at any visit. However, a statistically significant longitudinal change in distance stereopsis was observed in the DIMS group (p = 0.007).

**Table 7:** Evolution of Stereopsis according to lens type and viewing distance in myopic children.

Variable	Group	Basal	6m	12m	18m	p-valor Longitudinal
L-Stereo (”)	SV	59.53 ± 30.61	71.25 ± 37.22	60.67 ± 31.73	56.36 ± 28.03	0.775
	DIMS	81.25 ± 54.76	70.63 ± 91.25	49.38 ± 20.48	49.39 ± 28.02	**0.007**
	PAL	64.31 ± 40.64	61.54 ± 31.85	60.00 ± 32.25	53.00 ± 18.89	0.887
C-Stereo (”)	SV	35.25 ± 34.68	34.28 ± 17.35	35.90 ± 24.55	26.77 ± 13.78	0.926
	DIMS	43.72 ± 37.97	36.06 ± 16.21	26.63 ± 13.74	34.31 ± 30.84	0.078
	PAL	34.73 ± 24.39	33.69 ± 22.32	32.41 ± 25.02	30.10 ± 27.12	0.473

***Notes:*** *Values are expressed as mean ± standard deviation. D-Stereo and N-Stereo represent distance and near stereopsis, respectively, in arcseconds (”).*

## 4. Discussion

This study investigated the effect of different ophthalmic lens designs for myopia control in children, focusing on AL and SE as the main outcomes related to myopia progression, while also providing a comprehensive evaluation of accommodative function, binocular vision, and overall visual performance during an 18-month follow-up period. In this preliminary observational study, DIMS lenses were evaluated as a myopia control strategy, whereas PALs were included as a historical comparator [14], and SV lenses represented conventional correction. The interpretation of the findings should consider the non-randomized design and the baseline age differences between groups. The DIMS group was younger than both the SV (p = 0.043) and PALs groups (p = 0.001), while no differences were observed between SV and PALs. Since age is a relevant determinant of myopia progression, with younger children generally showing faster changes in SE and AL, these differences should be considered when interpreting between-group comparisons [31]. Similarly, baseline SE differed significantly among groups (p = 0.036), mainly due to greater myopia in the PALs group compared with SV according to Dunn’s post hoc test (p = 0.048). Therefore, the longitudinal analysis focused primarily on changes from baseline rather than absolute values at each follow-up visit.

The results showed that children wearing DIMS lenses presented the lowest SE progression over the 18-month follow-up, whereas differences between lens designs were less evident for AL. Friedman analyses of longitudinal changes in absolute values (Supplementary Tables S1 and S2), followed by Wilcoxon signed-rank post hoc comparisons, revealed significant differences among follow-up visits in all groups for both SE and AL. However, the temporal pattern of these changes differed among lens designs, with SV and PALs showing more pronounced early changes, particularly between baseline and 6 months, whereas DIMS showed a more stable progression pattern throughout follow-up.

Focusing on SE, the lower progression observed with DIMS is consistent with previous evidence. In the randomized clinical trial conducted by Lam et al. [15], including 183 Chinese children with a mean age of approximately 10 years, DIMS lenses resulted in an SE change of −0.31 D at 18 months compared with −0.72 D in the SV group. The refractive change observed in the DIMS group was therefore comparable to the value found in the present study (−0.30 D), whereas the progression in the SV group was lower than that reported by Lam et al. [15] (−0.59 D vs −0.72 D). This difference may partly be related to the older age of participants in the present cohort, particularly in the SV group (12.50 ± 2.76 years) [31]. Similarly, Nucci et al. [33] reported lower SE progression with DIMS compared with SV lenses in a European cohort, with changes of approximately −0.18 D and −1.10 D after 12 months, respectively. Although the difference between lens designs was larger than that observed in the present study, both studies showed a lower refractive progression in children wearing DIMS lenses compared with SV correction.

In contrast, the effect on AL was less evident. Lam et al. [15] reported a mean AL increase of approximately 0.15 mm in the DIMS group compared with 0.43 mm in the SV group at 18 months, whereas the present cohort showed more similar axial changes between lens designs. Similarly, Nucci et al. [33] observed significantly lower axial elongation with DIMS compared with SV lenses after 12 months, and suggested that differences in study population, sample size, follow-up duration, or treatment adherence may contribute to variability in the magnitude of the observed axial effects.

Regarding PALs, the myopia control effect observed in this study was limited, which is consistent with previous evidence. Gwiazda et al. [31] evaluated 469 children over three years using PALs with a + 2.00 D near addition and reported a cumulative SE progression of −1.28 D compared with −1.48 D in SV lenses, representing a mean difference of 0.20 ± 0.08 D. Although statistically significant (p = 0.004), this difference was considered clinically modest. In the present study, PALs incorporated a lower addition (+0.80 D), which may partly explain the limited effect observed, as previous studies have suggested that higher addition powers may provide greater myopia control effects [30,31]. Recent meta-analyses have also reported small and variable effects of PALs for myopia control compared with newer defocus-based lens designs [34].

Regarding accommodative function, no significant differences were found between groups across the variables studied, except for AF-B at baseline in a single condition. Therefore, no clinically relevant differences in accommodative variables were observed between lens designs during follow-up. Previous studies with DIMS and orthokeratology have reported improvements in accommodative facility over time, along with better accommodation–vergence coordination and slight reductions in accommodative lag [35].

When analyzing temporal changes across the seven accommodative variables evaluated for the three lens types, significant differences were observed at 18 months in only 5 of 21 possible comparisons, 3 of which corresponded to PALs. Of these 5 changes, three involved AA, and two were smaller than 0.5 D, thus not clinically significant. For instance, the change in binocular AA for DIMS lenses was 0.15 D. These results are consistent with the long-term study by Lam et al. [36], which evaluated monocular and binocular AA after six years of treatment across different lens-use sequences and found no significant differences between groups. In the present study, significant differences for DIMS lenses were observed only in binocular measurements at 18 months.

Similarly, Yahaya et al. [18], in a 24-month study of 25 Malaysian children wearing DIMS lenses, reported changes in accommodative function (AA-M, AA-B, NRA, PRA) measured every 6 months. Changes in NRA and PRA were not clinically significant, and binocular function remained stable, as in our study. They reported a 0.25 D reduction in accommodative lag at 12 months and an average annual reduction of 0.8 D in AA-M and AA-B [18]. In our data, AA-M and AA-B decreased at 6 and 12 months for all three lens types, recovering by 18 months, with no significant differences compared with baseline. Lam et al. [31] found an average annual reduction of 0.75 D in AA-M and AA-B and a 0.15 D accommodative lag over two years in both DIMS and SV groups. The recovery observed at 18 months in our study may reflect ethnic differences or sample size effects. Given our 18-month follow-up, we cannot determine longer-term trends. Overall, accommodative function appears to remain stable with DIMS use, although minor inter-study differences exist.

With respect to horizontal and vertical phorias at both distance and near, no significant differences were observed between lens types or over time within groups, consistent with previous reports [18,31,36]. No significant changes in distance or near phorias were observed over time in any study. It is worth noting that the baseline distance horizontal phoria showed a more esophoric tendency than generally reported in pediatric populations [37]. However, Jiménez et al. [38] also described a slight esophoric tendency in a large cohort of Spanish children, and the large variability observed in our sample suggests that this finding likely reflects sample-specific characteristics rather than a consistent binocular pattern.

Regarding fusional vergences, results showed general stability across conditions, with no clinically relevant differences between lens types. Baseline differences were observed in only two conditions (negative recovery at distance and near), which did not persist over time. Changes at 18 months were isolated and may reflect physiological variability. Yahaya et al. [18] reported no significant changes in NFV break or recovery, nor in PFV break, although some improvements in PFV were noted, suggesting enhanced capacity to maintain and recover binocular vision. Despite minor methodological differences, these findings suggest that binocular vision remained stable across the evaluated lens designs. Significant changes in near point of convergence were reported in earlier research [18], a parameter not assessed in the present study.

No significant between-group differences were found for distance or near stereopsis. Longitudinally, a meaningful improvement of approximately 31 arcseconds was observed at 18 months in distance stereopsis in the DIMS group. Yahaya et al. [18] reported a 10 arcsecond improvement in near stereopsis with DIMS, though considered not clinically significant. Lam et al. [39] reported no significant stereopsis changes. Improvements observed by Yahaya et al. [18] were explained by Erdinest et al. [40] as a dual mechanism involving motor adaptation and perceptual learning based on neural plasticity associated with DIMS use, which may represent a possible explanation.

Corrected distance and near visual acuity remained stable across lens types and over time, indicating preserved visual acuity throughout follow-up. These findings align with previous reports [16,36], who reported no significant changes in visual acuity. This overall stability is consistent with the broader evidence suggesting that high-contrast visual acuity is generally preserved with DIMS and other myopia-control optical designs, despite potential changes in other aspects of visual performance [15]. In contrast, other studies [18,39] reported improvements in distance and in the case of Yahaya et al. [18], also in near visual acuity, after two years of follow-up. In Western European populations, Nucci et al. [33] compared four groups (0.01% atropine, DIMS, combined DIMS + 0.01% atropine, and monofocal control) and reported significantly less visual acuity deterioration in treatment groups at 6 and 12 months, although numerical values were not specified. McCullough et al. [25] found no baseline differences in near or distance visual acuity between DIMS and SV groups in UK children.

Regarding CS, improvements with DIMS were observed at two spatial frequencies, and at one frequency for SV lenses, representing a partial improvement. Kaymak et al. [20] evaluated eight myopic subjects under various lens conditions (SV and DIMS) in central and peripheral vision (22° nasal and temporal) under photopic and mesopic conditions, with and without glare. No significant differences were found centrally between SV and DIMS lenses, although DIMS performed slightly worse peripherally. This laboratory-based short-term study reported preserved central visual performance with DIMS lenses, which is consistent with the pattern observed in the present cohort. Conversely, Li et al. [19], in 36 myopic children, reported a small but significant central high-frequency CS reduction with DIMS compared with aspheric lenses. Taken together, the present findings suggest that DIMS lenses were not associated with a deterioration in contrast sensitivity over time, although the observed improvements were frequency-specific rather than generalized across spatial frequencies. This is relevant because contrast sensitivity provides information about visual performance that is not captured by visual acuity alone.

No significant differences were found in VDI between lens types or over time. Given the multiple power zones incorporated into DIMS lenses, changes in discrimination performance might be expected due to differential illumination and the presence of peripheral microlenses [15]. However, VDI results demonstrated stable discrimination performance across the three lens types. To date, quantitative assessment of light disturbances has not been widely investigated in myopia-control optical interventions. Although quantitative measures of light disturbances and visual disturbances have been reported for other myopia-control optical designs, particularly dual-focus contact lenses [41], these metrics appear to have been less frequently evaluated in comparisons involving DIMS, PALs, and SV spectacles. In contrast, previous studies of DIMS have mainly focused on visual acuity, CS, accommodation, binocular function, and subjective visual symptoms [15]. Therefore, the absence of significant differences in VDI across the three lens types provides additional evidence that these optical designs were not associated with measurable differences in visual disturbances over the 18-month follow-up. This finding complements the contrast sensitivity and visual acuity results by providing a specific quantitative assessment of visual disturbances that may not be captured by conventional measures of visual performance.

Visual questionnaire outcomes also showed no significant between-group or longitudinal differences in reported symptoms, suggesting good subjective tolerance across lens types, including DIMS. These findings are consistent with previous reports showing no subjective visual deterioration [42,43].

This study has several limitations that should be considered when interpreting the results. First, the non-randomized design and allocation based on participant preference may have introduced selection bias and baseline differences between groups, particularly in age distribution. Second, the relatively small sample size may have reduced the ability to detect subtle differences, particularly in accommodative, binocular, and visual performance outcomes. The absence of an a priori sample size calculation should also be considered, and the findings require confirmation in larger prospective studies. Third, refractive error was characterized using a non-cycloplegic procedure, whereas other studies have used cycloplegic measurements [3,4,11,16]. Although fogging techniques and other precautions were used to minimize accommodative influence during subjective refraction, some residual accommodative effects cannot be completely excluded. This methodological difference should therefore be considered when comparing our findings with the existing literature.

In summary, this study should be considered an exploratory prospective observational investigation providing preliminary evidence on SE, AL, accommodative and binocular vision parameters, visual performance, and patient-reported outcomes in myopic Spanish children. The three groups should be interpreted as parallel observational cohorts, allowing the characterization of longitudinal changes in refractive, biometric, and visual outcomes associated with each lens design.

## 5. Conclusions

In conclusion, this study in Spanish children suggests that the use of DIMS lenses is associated with reduced myopia progression in terms of SE, whereas differences in AL between lens designs were less evident, although the magnitude and statistical strength of these findings may be influenced by sample size and study design limitations. Binocular and accommodative function remained generally stable over the 18-month follow-up, with DIMS lenses showing similar patterns of longitudinal change to PALs and SV lenses. Visual Visual function, assessed through CS and VDI, also remained stable, with no significant differences observed between lens designs, resulting in comparable outcomes across groups.

## Supporting information

S1 Table Longitudinal changes in absolute spherical equivalent (SE) values across follow-up visits. (DOCX)

S2 Table Longitudinal changes in absolute axial length (AL) values across follow-up visits. (DOCX)
