# Supplementary material for: Comparative longitudinal evaluation of DIMS, progressive addition and single-vision lenses on myopia progression, binocular and visual performance in Spanish children
Source: PLoS One. 2026 Oct 26;21(10):e0361471. doi: 10.1371/journal.pone.0361471 (PMC13704865; doi:10.1371/journal.pone.0361471)
Supplement: Supplementary file 1 [file pone.0361471.s001.docx]

# **Supporting information**

**S1 Table**. Longitudinal changes in absolute spherical equivalent (SE) values across follow-up visits.

| Group | Baseline | 6M | 12M | 18M | χ² | p |
| --- | --- | --- | --- | --- | --- | --- |
| SV | -1.75 ± 0.87 | -2.10 ± 0.88* | -2.23 ± 0.97 | -2.34 ± 1.07 | 14.87 | 0.002 |
| DIMS | -1.93 ± 1.67 | -2.05 ± 1.72 | -2.08 ± 1.71 | -2.23 ± 1.72 | 8.26 | 0.041 |
| PALs | -2.95 ± 1.54 | -3.48 ± 1.56* | -3.39 ± 1.48 | -3.46 ± 1.64 | 13.22 | 0.004 |

***Abbreviations:*** *SV, single-vision; DIMS, Defocus Incorporated Multiple Segments lenses; PALs, Progressive Addition Lenses. Values are presented as mean ± SD. Longitudinal analyses included participants with complete SE measurements across all four visits. Overall changes were assessed using the Friedman test; post hoc comparisons were performed using the Wilcoxon signed-rank test with Bonferroni correction (α = 0.0167). * indicates a significant difference from the preceding visit.*
