# Supplementary material for: Comparative longitudinal evaluation of DIMS, progressive addition and single-vision lenses on myopia progression, binocular and visual performance in Spanish children
Source: PLoS One. 2026 Oct 26;21(10):e0361471. doi: 10.1371/journal.pone.0361471 (PMC13704865; doi:10.1371/journal.pone.0361471)
Supplement: Supplementary file 2 [file pone.0361471.s002.docx]

# **Supporting information**

**S2 Table**. Longitudinal changes in absolute axial length (AL) values across follow-up visits

| Group | Baseline | 6M | 12M | 18M | χ² | p |
| --- | --- | --- | --- | --- | --- | --- |
| SV | 23.80 ± 0.69 | 24.01 ± 0.64 | 24.05 ± 0.64 | 24.12 ± 0.65* | 19.35 | <0.001 |
| DIMS | 23.63 ± 0.85 | 23.73 ± 0.98 | 23.78 ± 0.97 | 23.86 ± 1.02 | 12.95 | 0.005 |
| PALs | 24.77 ± 0.82 | 24.87 ± 0.83 | 24.96 ± 0.86* | 25.05 ± 0.86* | 22.44 | <0.001 |

***Abbreviations:*** *SV, single-vision; DIMS, Defocus Incorporated Multiple Segments lenses; PALs, Progressive Addition Lenses. Values are presented as mean ± SD. Longitudinal analyses included participants with complete AL measurements across all four visits. Overall changes were assessed using the Friedman test; post hoc comparisons were performed using the Wilcoxon signed-rank test with Bonferroni correction (α = 0.0167). * indicates a significant difference from the preceding visit.*
